# Single-cell transcriptomics reveals distinct microglial state remodeling associated with the (R)-nicotine/diosmetin combination and galantamine in LPS-challenged BV2 cells

**DOI:** 10.3389/fimmu.2026.1901868

**Published:** 2026-09-01

**Authors:** Yihan Liu, Hao Yu, Jingbin Zhang, Xin Li, Yunu Ma, Chunmei Guang, Wenjun Mu, Huan Chen, Hongwei Hou

**Affiliations:** 1 Beijing Life Science Academy, Beijing, China; 2 China National Tobacco Quality Supervision & Test Centre, Zhengzhou, China

**Keywords:** Batf-Atf3-associated transcriptional pattern, BV2 microglial-like cells, microglial state remodeling, neuroinflammation, single-cell RNA sequencing

## Abstract

**Background:**

Microglial neuroinflammation contributes to the progression of neurodegenerative diseases, yet it remains challenging to attenuate inflammatory responses while preserving cellular function. The effects of (R)-nicotine, diosmetin, their combined administration, and galantamine on heterogeneous BV2 transcriptional states have not been compared at single-cell resolution.

**Methods:**

LPS-stimulated BV2 microglial-like cells were treated with (R)-nicotine, diosmetin, their combination (DR), or galantamine. Single-cell RNA sequencing was performed with three biological replicates per group and integrated with RNA velocity and SCENIC regulon inference to characterize treatment-associated state redistribution, inferred local transcriptional directionality and regulon-activity patterns. Functional validation included CCK-8 metabolic activity assays, multiplex cytokine ELISA, BDNF/GDNF quantification, qPCR, and high-content immunofluorescence analysis of iNOS and Arg1 at single-cell resolution.

**Results:**

LPS decreased the relative abundance of the *Itgae^+^/Plk4^+^* cluster while increasing the *Nmur1^+^/Limk2^+^* cluster and inflammatory effector programs. DR treatment suppressed pro-inflammatory cytokine release without significantly reducing CCK-8-assessed metabolic activity, increased the *Itgae^+^/Plk4^+^* cluster proportion, and increased BDNF/GDNF relative to LPS. RNA velocity and SCENIC analyses suggested that DR and galantamine showed distinct transcriptional and regulatory patterns: DR attenuated Batf-associated inflammatory regulons and was associated with increased Atf3-linked stress-response activity, whereas galantamine preferentially engaged DNA repair and genome-maintenance programs.

**Conclusion:**

These findings indicate that the DR condition was associated with remodeling of LPS-challenged BV2 microglial-like states, attenuation of inflammatory programs, and increased neurotrophic outputs relative to LPS, without significantly reducing CCK-8-assessed metabolic activity. This study provides a single-cell characterization of distinct treatment-associated responses to (R)-nicotine, diosmetin, their combined administration, and galantamine.

## 1 Introduction

Microglia, the resident macrophages of the central nervous system (CNS), perform essential homeostatic functions including immune surveillance, synaptic pruning, debris clearance, and tissue repair (1, 2). However, when their activation becomes maladaptive and sustained, these cells can perpetuate chronic neuroinflammation, secrete neurotoxic factors (3), and ultimately drive the progression of various neurodegenerative diseases such as Alzheimer’s disease (AD), Parkinson’s disease (PD), and multiple sclerosis (MS). Although these are distinctly chronic conditions, the lipopolysaccharide (LPS)-induced acute neuroinflammation model remains extensively utilized as a widely used experimental model. By reliably triggering the core mechanistic pathways of microglial hyperactivation, immune metabolic shifts, and the release of pro-inflammatory mediators, the LPS model reliably recapitulates core inflammatory events that parallel or exacerbate neurodegeneration, making microglial hyperactivation a tractable target for therapeutic intervention. While the classical “M1/M2” dichotomy provided a foundational framework for understanding microglial activation, recent advances in single-cell transcriptomics have greatly refined this view (4, 5). It is now widely recognized that microglial “activation” is not a simple binary switch but a dynamic and continuous spectrum of functional states, shaped by their ontogeny, the specific tissue microenvironment, and distinct pathological stimuli. In the CNS, microglia maintain physiological homeostasis through a conserved set of genes known as the “sensome,” but during neurodegenerative conditions like AD, they adopt distinct disease-associated molecular patterns (DAMs) or injury-associated states, which are closely linked to disease progression (6–10).

A critical challenge in neuroinflammatory research is identifying interventions that attenuate inflammatory responses without broadly compromising microglial functions. Cholinergic signaling has been implicated in immune regulation through the cholinergic anti-inflammatory pathway (CAP) (11–13). Among nicotinic receptor subtypes, the α7 nicotinic acetylcholine receptor (α7nAChR) has been proposed as one potential contributor to inflammatory regulation in microglial and other immune-cell models (14–16). Galantamine has also been reported to modulate inflammatory responses in several experimental contexts (17, 18). However, the receptor mechanisms underlying these effects are context dependent and cannot be inferred solely from the pharmacological identity of the administered compounds. These pharmacological observations provided background for compound selection but do not establish α7nAChR involvement in the present BV2 model.

In parallel, bioactive flavonoids have been investigated as modulators of inflammatory and oxidative-stress responses (19, 20). Diosmetin, a naturally occurring flavonoid, has been reported to affect Nrf2–Keap1/NF-κB- and NLRP3-related signaling in experimental models (19, 20). (R)-Nicotine has also received increasing pharmacological attention as a nicotine stereoisomer, with previous studies examining its stereochemical properties and potential differences in abuse-related effects (21–23). Separately, Meng et al. identified diosmetin as a candidate α7nAChR positive allosteric modulator through computational screening and calcium-signaling assays (24). However, this prior observation does not establish receptor-subtype selectivity, α7nAChR dependence, or a receptor-level interaction in BV2 cells. Therefore, in the present study, (R)-nicotine and diosmetin were evaluated both individually and in combination, with galantamine included as a pharmacological comparator, to characterize treatment-associated differences in inflammatory outputs, CCK-8-assessed metabolic activity, and marker-defined BV2 transcriptional states. The study was designed as a comparative treatment-response analysis rather than a test of pharmacological synergy or α7nAChR-dependent signaling.

Because broad suppression or depletion of microglia may disrupt CNS homeostasis (25–27), it is relevant to determine whether the tested treatments can attenuate inflammatory programs without reducing CCK-8-assessed metabolic activity (4, 28). In the present study, we used single-cell RNA sequencing and complementary functional assays to compare the responses of LPS-challenged BV2 cells to (R)-nicotine, diosmetin, their combined administration (DR), and galantamine. RNA velocity and SCENIC analyses were used to characterize treatment-associated differences in marker-defined state distributions, inferred local transcriptional directionality, and regulon activity (29–31). We hypothesized that the different treatment conditions would be associated with distinguishable transcriptional and functional response patterns in LPS-challenged BV2 cells.

## 2 Materials and methods

### 2.1 Cell culture and treatment

BV2 murine microglial cells were purchased from Procell Life Science & Technology (Wuhan, China; Cat# CL-0493A) and maintained in DMEM supplemented with 10% fetal bovine serum and 1% penicillin–streptomycin at 37 °C in a humidified incubator with 5% CO2. Cells were seeded one day prior to stimulation. Inflammation was induced with LPS (1 μg/mL). Treatment groups included vehicle control, LPS alone, galantamine (GAL), diosmetin (DIO), (R)-nicotine (R), and the combination of (R)-nicotine plus diosmetin (DR). Diosmetin (DIO) was used at 10/25 μM, (R)-nicotine (R) at 10 μM, and the combination group (DR) was treated with 10 μM DIO plus 10 μM R. Galantamine was used at 10 μM. An additional DR+MLA group received methyllycaconitine (MLA, 10 μM) together with DR during the pretreatment period. Unless otherwise stated, cells were pretreated with the indicated compounds for 4 h. The pretreatment medium was then removed, cells were washed with PBS, and fresh medium containing LPS (1 μg/mL) was added for 24 h without the test compounds. The abbreviations used throughout the text and figures were CON (vehicle control), LPS (lipopolysaccharide), GAL (galantamine), DIO (diosmetin), R [(R)-nicotine], and DR [(R)-nicotine plus diosmetin]. Samples for scRNA-seq, ELISA, qPCR, and immunofluorescence were collected 24 h after LPS exposure. For the CCK-8 assay, CCK-8 reagent was added at the experimental endpoint and incubated for 2 h before absorbance measurement.

### 2.2 Cellular metabolic activity and cytokine quantification

Cellular metabolic activity was evaluated using a Cell Counting Kit-8 assay (CCK-8; Dojindo Laboratories, Japan). At the experimental endpoint, CCK-8 reagent was added and cells were incubated for 2 h before absorbance was measured at 450 nm.

For the quantification of secreted factors, culture supernatants were collected 24 h after LPS exposure and clarified by centrifugation to remove cellular debris. IL-6, IL-1β, TNF-α, MCP-1, IL-17, BDNF and GDNF were measured using mouse ELISA kits from Shanghai Jianglai Biotechnology Co., Ltd. (Shanghai, China). Manufacturer and catalog-number information is provided in **Supplementary Table 4A**.

Samples were diluted 1:10 for IL-6, 1:5 for IL-1β, TNF-α, and MCP-1, and 1:2 for IL-17, BDNF, and GDNF. Absorbance was measured at 450 nm, and analyte concentrations were calculated using four-parameter logistic standard curves. All assays were performed according to the manufacturers’ instructions. Each group contained three independent biological replicates, and each biological sample was measured in technical duplicate. Technical duplicates were averaged before statistical analysis and were not treated as independent observations.

### 2.3 Immunofluorescence and quantification

Cells were fixed with 4% paraformaldehyde, permeabilized with 0.1% Triton X-100, blocked with 5% bovine serum albumin, and incubated with primary antibodies against *iNOS* and *Arg1*, followed by fluorescent secondary antibodies. Nuclei were counterstained with DAPI. Plates were imaged on a high-content screening platform. Single-cell segmentation was performed using nuclear masks expanded to approximate cellular regions. Quantification was reported as per-cell mean fluorescence intensity (MFI) for each marker, exported directly from the instrument analysis software. For each well, multiple fields were acquired and aggregated to generate one value per biological replicate.

### 2.4 Quantitative real-time PCR

To evaluate treatment-associated marker changes in microglia, quantitative real-time PCR (qPCR) was performed. Relative mRNA expression levels of inflammation- and activation-associated markers (*iNOS*, *Cd80*, *Cd86*), activation/phagolysosomal and scavenger-receptor-associated markers (*Cd68*, *Cd163*), and key transcription factors (*Batf*, *Atf3*) were quantified by qPCR to assess treatment-induced phenotypic changes and corroborate SCENIC-derived regulon inferences. These markers were interpreted individually and were not used to assign binary M1/M2 states. *Il1b, Il6*, and *Tnf* expression was additionally quantified in the CON, LPS, DR, and DR+MLA groups to determine whether MLA co-treatment altered the DR-associated expression pattern. Total RNA was extracted using a Universal Total RNA Extraction Kit, and cDNA was synthesized using an Evo M-MLV reverse-transcription premix containing a genomic-DNA removal step (Accurate Biotechnology, Hunan, China). Quantitative PCR was performed using a SYBR Green Pro Taq HS premix on a Roche LightCycler 480 instrument.

The amplification program consisted of initial denaturation at 95 °C for 30 s, followed by 40 cycles of denaturation at 95 °C for 5 s and annealing/extension at 60 °C for 30 s. A dissociation-curve analysis was performed after amplification. Relative mRNA expression was calculated using the 2^−ΔΔCt method with Actb as the reference gene. Each biological sample was measured in technical duplicate, and duplicate Ct values were averaged before calculation and statistical analysis. Primer sequences and qPCR reagent details are provided in **Supplementary Table 4B**.

### 2.5 Cell preparation

After cell collection, adherent BV2 cells were washed in ice-cold PBS (Hyclone SH30256.01) and dissociated using Trypsin (Sigma SH30042.01). DNase I (Sigma 9003-98-9) treatment was optional according to the viscosity of the homogenate. Cell count and viability were estimated using SeekMate Tinitan Fluorescence Cell Counter (SeekGene M002C) with AO/PI reagent with optional removal of debris and dead cells (Miltenyi 130-109-398/130-090-101). Finally, fresh cells were washed twice in the RPMI1640 (Gibco 11875119) and then resuspended at 1×106 cells per ml in RPMI1640 and 2% FBS (Gibco 10100147C).

### 2.6 Single cell RNA-seq library construction and sequencing

Single-cell RNA-Seq libraries were prepared using SeekOne^®^ DD Single Cell 3’ library preparation kit (SeekGene Catalog No. K00202). Briefly, an appropriate number of cells were mixed with reverse transcription reagent and then added to the sample well in SeekOne^®^ chip S3. Subsequently Barcoded Hydrogel Beads (BHBs) and partitioning oil were dispensed into corresponding wells separately in chip S3. After emulsion droplet generation reverse transcription was performed at 42°C for 90 minutes and inactivated at 85°C for 5 minutes. Next, cDNA was purified from broken droplets and amplified in PCR reaction. The amplified cDNA product was then cleaned, fragmented, end repaired, A-tailed and ligated to sequencing adaptor. Finally, the indexed PCR was performed to amplify the DNA representing 3’ polyA part of expressing genes which also contained Cell Barcode and Unique Molecular Index. The indexed sequencing libraries were cleaned up with VAHTS DNA Clean Beads (Vazyme N411-01), analyzed by Qubit (Thermo Fisher Scientific Q33226) and Bio-Fragment Analyzer (Bioptic Qsep400). The libraries were then sequenced on illumina NovaSeq X Plus with PE150 read length. Across the 18 single-cell libraries, 6.312 billion raw reads and 6.295 billion clean reads were generated. The mean sequencing depth ranged from 19,771 to 31,699 reads per cell. Median detected genes ranged from 2,019 to 2,882 per cell, and median UMI counts ranged from 6,369 to 10,274 per cell. Sequencing saturation ranged from 38.90% to 49.80%, and the proportion of reads mapped confidently to the genome ranged from 75.56% to 80.75%. Complete per-sample sequencing and library quality-control metrics are provided in **Supplementary Tables 6A, B**.

### 2.7 Seurat analysis

Downstream analyses were conducted in Seurat. The 18 biological samples were initially processed independently. Cells were retained if they expressed 200–6,000 detected genes, contained 3,000–25,000 UMIs, and had <5% mitochondrial reads. After sample-wise quality control, each sample was randomly down sampled to a maximum of 5,000 cells using a fixed random seed (32), yielding a balanced final dataset of 90,000 cells across 18 biological samples.

SCTransform normalization was performed independently for each sample (33, 34), after which the sample objects were merged while preserving sample identity. Visual inspection of the merged low-dimensional embedding colored by sample identity did not reveal dominant sample-specific segregation. Therefore, no additional batch-correction procedure, such as Harmony or Seurat anchor-based integration, was applied, and treatment identity was not treated as a batch covariate. Cell-cycle scores were calculated using Seurat::CellCycleScoring, and S.Score and G2M.Score were regressed during subsequent SCTransform normalization.

Principal component analysis was performed on the SCT assay. Neighborhood graphs were constructed using the first 12 principal components, clustering was performed at resolution = 0.4, and UMAP was generated using dimensions 1–12 with n.neighbors = 20. Cluster markers were identified using FindAllMarkers with default settings unless otherwise specified.

No dedicated computational doublet-detection algorithm was applied. Potential doublets were minimized through the upper feature-count and UMI-count thresholds, mitochondrial-read filtering, and sample-wise quality control before balanced down sampling. A complete summary of the sample-wise preprocessing, quality-control, normalization, merging, cell-cycle regression, and doublet-handling workflow is provided in **Supplementary Table 6C**.

### 2.8 Gene module scoring and state classification

**Figure 1:**
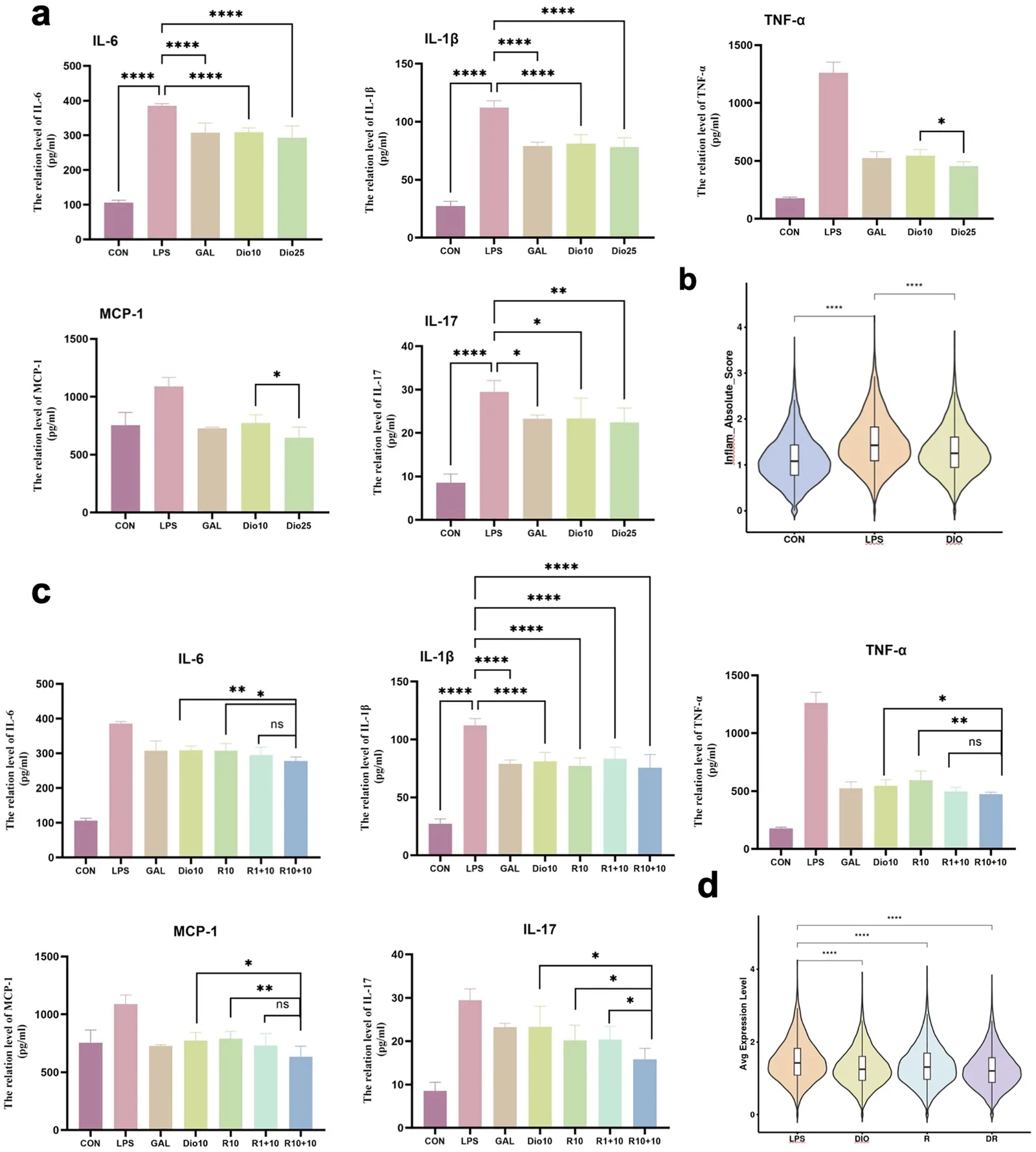
Effects of (R)-nicotine and diosmetin on LPS-induced inflammatory readouts in BV2 cells. **(a)** ELISA analysis of *IL-6*, TNF-α, *IL-1β*, *MCP-1*, and *IL-17* secretion in LPS-induced BV2 cells treated with Diosmetin (10, 25 μM), showing dose-dependent inhibition. **(b)** Violin plots showing the single-cell “Inflammation Score” across groups. The Diosmetin (DIO) group exhibited a lower median score compared to the LPS group. **(c)** Evaluation of combined effects. The combination of R-Nicotine (10μM) and Diosmetin (10μM) (R10 + 10, DR combination treatment) significantly further reduced *IL-17* and *MCP-1* levels compared to monotherapies. **(d)** Violin plots of the “Inflammation Score” for combination treatments, showing a reduction in the inflammatory transcriptional program under the DR condition. Data are presented as mean ± SD. Statistical comparisons were performed using ordinary one-way ANOVA followed by Tukey’s multiple-comparisons test. Horizontal brackets indicate the specific treatment comparisons. Each group contained three independent biological replicates (n = 3). ELISA technical duplicates were averaged within each biological replicate before statistical analysis. Cell-level distributions are shown in the violin plots, whereas statistical inference was based on biological-replicate-level summaries. *P < 0.05, **P < 0.01, ****P < 0.0001.

**Figure 2:**
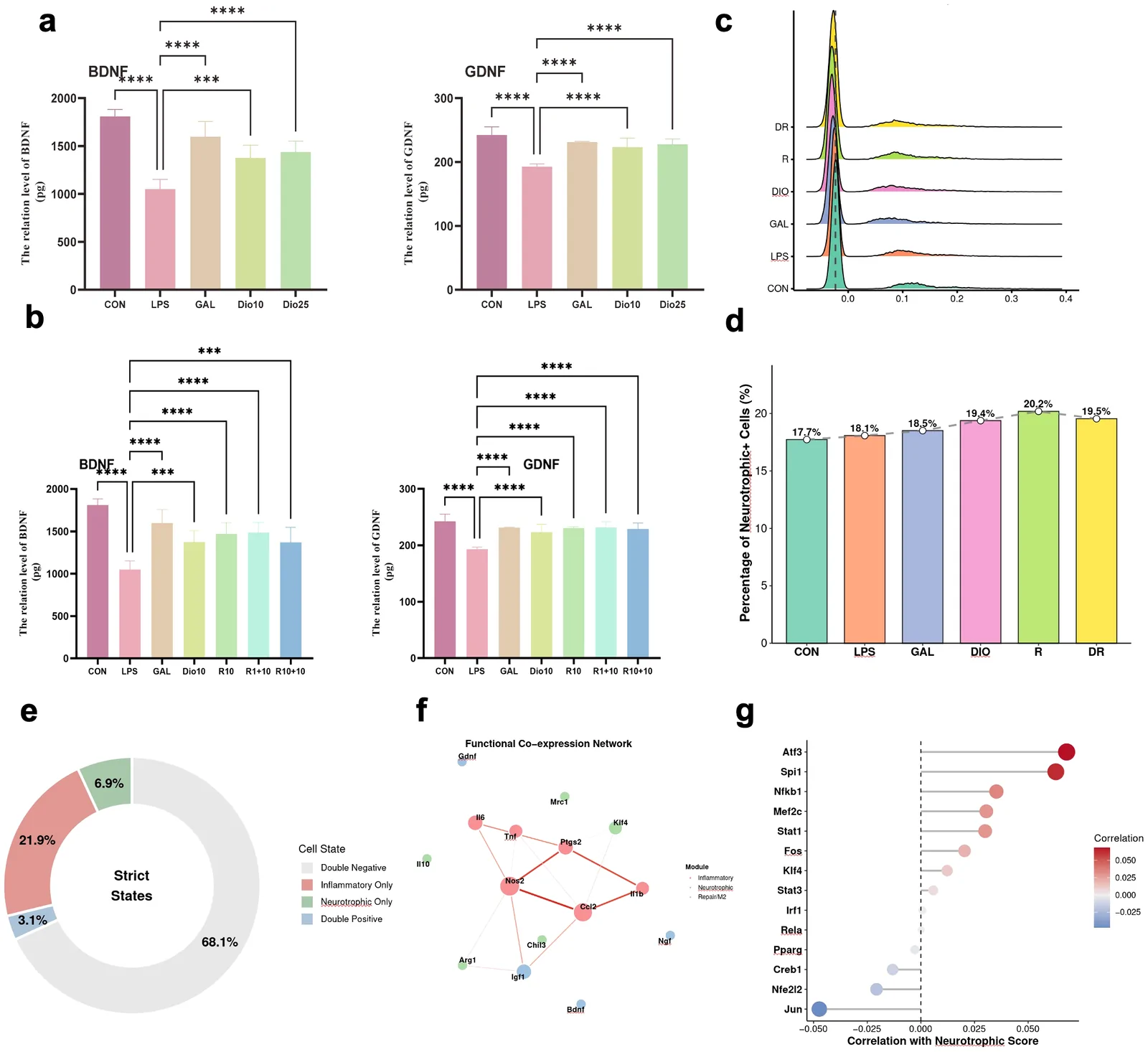
Restoration of neurotrophic function accompanied by changes in neurotrophic-score distributions. **(a, b)** ELISA quantification of neurotrophic factors BDNF **(a)**, left) and GDNF (a, right) for Diosmetin dose-response, and BDNF **(b)**, left) and GDNF **(b)**, right) for combination therapy. The combination therapy (R10 + 10) significantly increased BDNF and GDNF levels relative to the LPS group of these factors. **(c)** Ridge plot of the single-cell “Neurotrophic Score”. Drug treatment induced a “functional long-tail” distribution. **(d)** Bar plot showing the percentage of cells with neurotrophic scores >0.05 across biological replicates. **(e)** Donut chart illustrating functional-state proportions among cells pooled across all six conditions (CON, LPS, GAL, DIO, R, and DR). **(f)** Functional co-expression network analysis identifying *Igf1* as a bridge node connecting inflammatory (*Nos2*, *Tnf*) and neurotrophic (*Bdnf*, *Arg1*) modules. **(g)** Dot plot showing the correlation between transcription factor activity and Neurotrophic Score, with Atf3, Jun, and Igf1 emerging as candidate regulators or network-associated nodes linked to neurotrophic-state enrichment. Statistical comparisons in panels **(a, b, d)** were performed using ordinary one-way ANOVA followed by Tukey’s multiple-comparisons test. Each group contained three independent biological replicates (n = 3). ELISA technical duplicates were averaged within each biological replicate before statistical analysis, and neurotrophic-positive fractions were calculated independently for each biological sample. **(c, e–g)** are descriptive single-cell distribution or network visualizations and were not treated as independent biological replicates. Data are presented as mean ± SD. *P < 0.05, ***P < 0.001, ****P < 0.0001.

To quantify functional programs at single-cell resolution, gene-module scores were calculated using Seurat::AddModuleScore. The inflammation score shown in **Figure 1** was calculated using *Ccl4, Nfkbia, Ccl2*, and *Ccl7*, whereas the neurotrophic score shown in **Figures 2c, d** was calculated using *Bdnf, Gdnf, Ngf, Ntf3, Igf1*, and *Fgf2*. Additional curated signatures representing microglial homeostasis, inflammatory/NF-κB activity, stress/integrated stress response, and proliferation/cell-cycle activity were calculated using Seurat::AddModuleScore and summarized within each sample–cluster combination.

The proliferation/cell-cycle signature was used for functional characterization of the marker-defined clusters and was distinct from the S.Score and G2M.Score covariates generated using Seurat::CellCycleScoring and regressed during SCTransform normalization. Neurotrophic-positive cells were defined using an empirical fixed threshold on the neurotrophic module score (score > 0.05). Neurotrophic module scores were primarily interpreted as continuous measurements, whereas the fixed score >0.05 cutoff was used descriptively to identify a broader upper-tail category. Because the sample-specific GMM-derived boundaries ranged from 0.1016 to 0.1697, the highest-mean GMM component was interpreted as a more stringent subset of the upper score distribution. To examine the upper neurotrophic-score distribution under an alternative operational definition, a three-component Gaussian mixture model was fitted independently to each biological sample. This GMM-based classification was treated as a descriptive alternative to, rather than a validation of, the fixed score >0.05 definition. Components were ordered by their mean scores, and cells with a posterior probability >0.5 of belonging to the highest-mean component were classified as GMM-defined score-high cells. The corresponding sample-specific cutoff was defined at the score where the posterior probability of the highest-mean component equaled 0.5. Module-score summaries and neurotrophic-positive fractions were calculated separately for each biological sample before group-level comparisons.

For the pooled four-state classification shown in **Figure 2e**, an absolute inflammation score was calculated as the mean normalized expression of *Ccl4*, *Nfkbia, Ccl2*, and *Ccl7*, whereas an absolute neurotrophic score was calculated as the mean normalized expression of *Bdnf, Gdnf, Ngf, Ntf3*, and *Igf1*. The inflammation and neurotrophic scores were dichotomized using the 75th and 90th percentiles, respectively, calculated across cells pooled from all six experimental conditions (CON, LPS, GAL, DIO, R, and DR). These thresholds yielded inflammation-prioritized, neurotrophic-prioritized, double-positive, and double-negative categories. Thus, *Fgf2* was included in the AddModuleScore-derived neurotrophic score used in **Figures 2c, d** but was not included in the absolute-expression score used for the pooled four-state visualization in **Figure 2e**.

Detection summaries and sample–cluster scores for the four curated signatures are provided in **Supplementary Table 2** and **S3**, respectively. Complete gene lists, scoring methods, and threshold definitions for the figure-specific inflammation, neurotrophic, absolute-expression, and GMM analyses are provided in **Supplementary Table 5**.

### 2.9 RNA velocity analysis

RNA velocity was computed using scVelo (v0.3.3) with the dynamical model. After velocity-specific quality control and cell matching, 34,882 cells were retained for RNA velocity analysis. Spliced and unspliced transcript counts accounted for 89.53% and 10.47%, respectively, of the combined spliced and unspliced counts. Genes were filtered (min_shared_counts=20) and the top 2000 highly variable genes were selected. Moments were calculated using 30 PCs and 30 neighbors. Velocity vectors were projected onto the UMAP embedding. Velocity confidence was calculated using scVelo as an additional quality metric. Unsupervised latent time was calculated as a complementary measure of inferred transcriptional ordering and placed *Nmur1^+^/Limk2^+^* cells within an early region of the inferred ordering. To qualitatively assess replicate-level consistency, velocity fields were additionally generated independently for the three DR biological replicates. The replicate-specific velocity objects contained 1,828, 1,907, and 1,873 cells, respectively, and showed comparable local vector organization. Replicate-level DR velocity maps are provided in **Supplementary Figure 4**, and the corresponding methodological details are summarized in **Supplementary Table 7**.

### 2.10 Regulon inference and co-expression network analysis

Transcription factor regulatory networks were inferred using pySCENIC (v0.12.1). Co-expression modules were identified with GRNBoost2 and pruned using cisTarget with mm9 reference databases (500bp upstream and TSS ±10kb). The default NES threshold of 3.0 was applied. Regulon activity for each cell was scored using the AUCell algorithm. For group-level comparisons, regulon AUC values were averaged within each biological sample. Differential regulon activity across conditions was used to identify candidate TFs associated with LPS-induced stress/inflammatory programs and treatment-associated remodeling (35, 36). For neurotrophic-associated analyses, co-expression networks were constructed around Bdnf, Gdnf, and related genes. Atf3- and Jun-associated regulon activities and Igf1 expression were evaluated for their associations with neurotrophic module scores. These analyses were interpreted as regulatory or network associations rather than evidence of causal upstream control.

### 2.11 Statistical analysis

Statistical analyses were performed in GraphPad Prism and R. For multi-group comparisons of CCK-8, ELISA, qPCR, immunofluorescence, cell-state proportions, biological-replicate-level module-score summaries, regulon activities, and neurotrophic-positive fractions, ordinary one-way ANOVA followed by Tukey’s multiple-comparisons test was used. Overall differences in the four curated signature scores across the nine marker-defined clusters were assessed using Friedman tests with biological sample identity as the blocking factor. P values were adjusted across the four signature-level tests using the Benjamini–Hochberg method.

Cell-state proportions, module-score summaries, regulon activities, and neurotrophic-positive fractions were calculated per biological replicate (n = 3 per group) prior to group-level comparison to avoid pseudo-replication. ELISA and qPCR technical duplicates were averaged within each biological replicate before statistical analysis and were not treated as independent observations. For immunofluorescence analyses, multiple imaging fields were aggregated to generate one value per biological replicate.

Violin plots, ridge plots, UMAPs, heatmaps, RNA velocity maps, donut charts, and network plots display cell-level or descriptive distributions. Cells were not treated as independent biological replicates; inferential comparisons were based on biological-replicate-level summaries. Horizontal brackets in the figures indicate the specific treatment groups included in each Tukey comparison. Data are presented as mean ± SD unless otherwise stated. P < 0.05 was considered statistically significant. A panel-by-panel summary of the statistical tests, sample sizes, and statistical units is provided in **Supplementary Table 8**.

## 3 Results

### 3.1 Single-cell atlas reveals drug-specific state shifts

**Figure 3:**
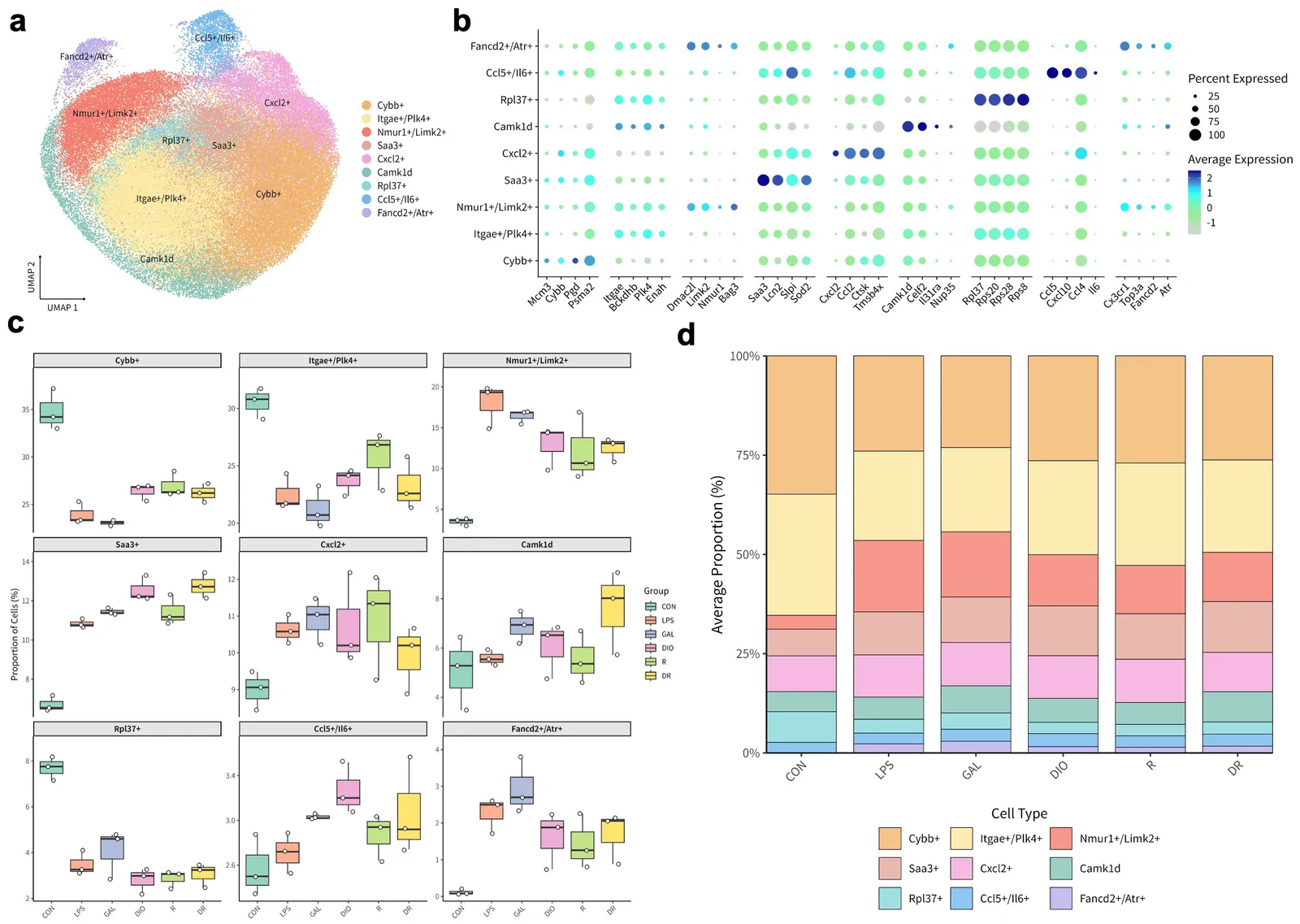
Single-cell transcriptomic atlas reveals treatment-associated redistribution of marker-defined BV2 states. **(a)** Uniform Manifold Approximation and Projection (UMAP) visualization of 9 distinct microglial clusters identified from scRNA-seq analysis. **(b)** Dot plot showing representative marker genes for the *Itgae^+^/Plk4^+^, Nmur1^+^/Limk2^+^, Saa3^+^, Cxcl2^+^, Ccl5^+^/Il6^+^*, and other marker-defined states. **(c)** Box plots illustrating the proportions of specific cell subpopulations across different treatment groups. **(d)** One-hundred-percent stacked bar plot showing cell-state composition across the independent treatment groups. LPS stimulation decreased the Itgae^+^/Plk4^+^ cluster and increased the *Saa3^+^* and Cxcl2^+^ clusters (*Saa3^+^, Cxcl2^+^)*. The combination treatment (DR) was associated with partial attenuation of this LPS-associated compositional change; while it had a limited effect on reducing the *Saa3^+^* cluster, it effectively increased the *Itgae^+^/Plk4^+^* cluster proportion and was associated with a lower relative abundance of the *Nmur1^+^/Limk2^+^* cluster than observed under LPS alone. Statistical comparisons of cell-state proportions in panel c were performed using ordinary one-way ANOVA followed by Tukey’s multiple-comparisons test. Data represent three independent biological replicates per group (n = 3). Panel **(d)** is a descriptive composition plot and was not used as an independent statistical test.

To resolve BV2 microglial-like state heterogeneity across LPS challenge and the different treatment conditions, we profiled cells from control, LPS, and treatment conditions using single-cell RNA sequencing. Unsupervised clustering identified nine transcriptionally distinct marker-defined states with proliferative, stress-responsive and inflammatory effector programs (**Figure 3a**). Marker-gene expression supported the nomenclature of these states (**Figure 3b**). Notably, these transcriptional states remained distinguishable after regression of cell-cycle scores during normalization, suggesting that the observed heterogeneity was not driven solely by proliferative differences. Cluster 1 showed elevated *Itgae* and *Plk4* expression, defining the *Itgae^+^/Plk4^+^* marker-based state, whereas the *Nmur1*^+^/*Limk2*^+^ cluster exhibited relatively elevated stress/ISR-associated activity. Additional clusters were enriched for inflammatory chemokine and stress-response signatures, including *Cxcl2*^+^, *Saa3*^+^, *Ccl5*^+^, and *Il6*^+^ programs, consistent with inflammatory effector-associated states.

Importantly, compositional analyses performed per biological replicate (n = 3 per group) showed that LPS was associated with a marked shift in state distribution (**Figures 3c, d**). The *Itgae*^+^/*Plk4*^+^ cluster proportion decreased, while the *Nmur1*^+^/*Limk2*^+^ cluster proportion increased substantially. Among all clusters, these two states exhibited the most consistent and intervention-sensitive changes, prompting us to use the relative representation of the *Nmur1*^+^/*Limk2*^+^–*Itgae*^+^/*Plk4*^+^ clusters as a descriptive framework for subsequent analyses. In contrast, several inflammatory effector-associated clusters (e.g., *Saa3*^+^ and *Cxcl2*^+^) did not uniformly contract under treatment, showing more limited compositional responses to treatment. Accordingly, downstream transcriptional and regulatory analyses focused on these marker-defined states.

Functional enrichment of cluster-specific markers further supported this annotation. The *Itgae*^+^/*Plk4*^+^ state was associated with pathways linked to cell interactions and structural regulation, whereas the *Nmur1*^+^/*Limk2*^+^ state showed enrichment for stress-adaptive and remodeling pathways (e.g., ubiquitin-mediated proteolysis and endocytosis-related programs), consistent with stress-adaptive and remodeling programs. These analyses framed a state-level hypothesis: LPS alters the relative distribution of the *Itgae^+^/Plk4^+^*, *Nmur1^+^/Limk2^+^*, and inflammatory effector-associated clusters, while the treatment conditions were associated with changes in the relative representation of these marker-defined states.

### 3.2 Secreted factors support immunomodulation without reduced metabolic activity

Prior to evaluating the inflammatory outputs, cellular metabolic activity was assessed using a CCK-8 assay. None of the tested drug concentrations significantly reduced the CCK-8 signal, which remained above 90% of the control level. (**Supplementary Figure S1**). Consequently, we next quantified inflammatory outputs to connect state remodeling to functional endpoints. LPS significantly increased secretion of multiple pro-inflammatory mediators, including *IL-6*, *IL-1β*, TNF-α, *MCP-1*, and *IL-17* (**Figure 1a**). Among these readouts, DR produced stronger suppression of *IL-17* and *MCP-1* than either single-agent treatment.

At the transcriptomic level, an inflammation gene-module score increased substantially under LPS and was reduced by treatment, with DR showing a consistent downward shift in score distribution (**Figures 1b–d**). Together, these data indicate that the combination does not merely affect a single cytokine pathway but dampens coordinated inflammatory transcriptional programs. Recalling our compositional observation (**Figure 3d**) that inflammatory effector-associated clusters showed variable compositional responses, these results are most parsimoniously explained by a combination of (i) suppression of inflammatory gene programs within marker-defined states and (ii) compositional rebalancing along the *Nmur1*^+^/*Limk2*^+^–*Itgae*+ axis, which collectively lowers the global inflammatory load.

### 3.3 Phenotypic validation and RNA velocity mapping of treatment-associated transcriptional patterns

**Figure 4:**
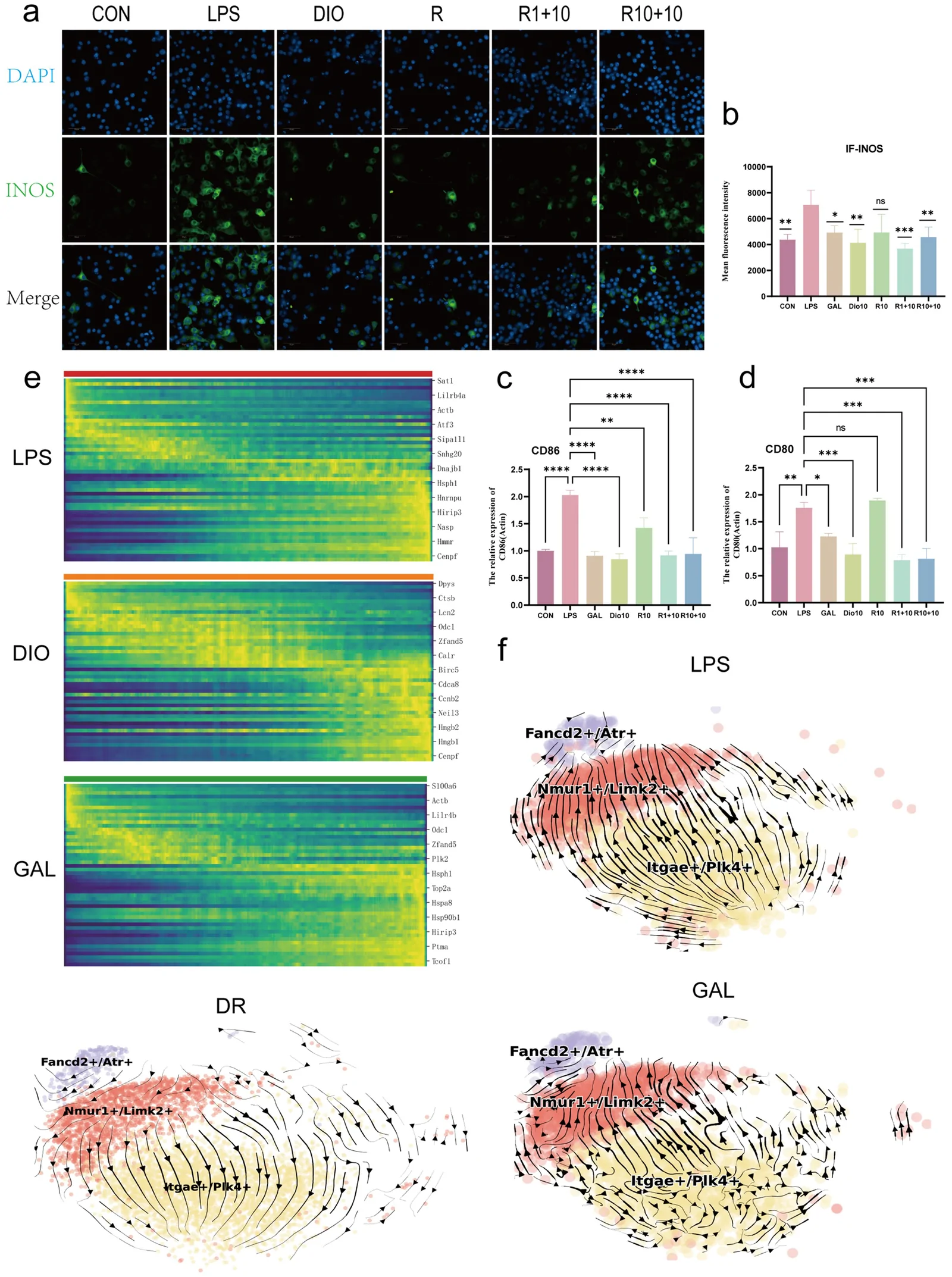
RNA velocity analysis reveals treatment-associated transcriptional and velocity patterns**. (a-d)** Immunofluorescence images **(a)** and quantification **(b)** of *iNOS* (inflammation-associated enzyme, green). qPCR analysis of activation-associated co-stimulatory markers *CD80* **(c)** and *CD86* **(d)** showing reduced expression of pro-inflammatory markers in the DR group. **(e)** Heatmap of differentially expressed genes (DEGs) across LPS, DIO, and GAL groups, highlighting distinct transcriptional profiles. **(f)** RNA velocity streamlines projected onto the UMAP embedding for LPS, DR, and GAL groups. In the LPS group, velocity vectors are directed toward inflammatory effector-associated clusters (Cxcl2^+^, Saa3^+^), indicating an inflammation-associated velocity pattern. DR and related combination treatments were associated with velocity vectors showing greater orientation toward Itgae^+^/Plk4^+^-enriched regions, consistent with a feedback- and stress-adaptation-associated velocity pattern. By contrast, GAL treatment showed preferential vector orientation toward DNA repair/defense gene-enriched regions, indicating a distinct genome-maintenance/repair-associated program. Statistical comparisons in panels b–d were performed using ordinary one-way ANOVA followed by Tukey’s multiple-comparisons test. Each group contained three independent biological replicates (n = 3). Multiple imaging fields were aggregated to generate one MFI value per biological replicate, and qPCR technical duplicates were averaged before statistical analysis. Panels e and f are descriptive transcriptomic and RNA velocity visualizations. *P < 0.05, **P < 0.01, ***P < 0.001, ****P < 0.0001, as applicable.

To connect transcriptomic state changes to functional phenotypes, we first validated the attenuation of the pro-inflammatory effector program at the protein and transcript levels, then mapped the underlying transcriptional dynamics using RNA velocity. High-content immunofluorescence imaging revealed that LPS stimulation induced marked accumulation of iNOS (an inflammation-associated nitric oxide-producing enzyme), producing a strong pro-inflammatory phenotype (**Figure 4a**). Quantification of the mean fluorescence intensity (MFI) showed that while single-agent treatments (GAL, DIO, or R) exerted partial inhibition, the DR combination therapy was associated with a pronounced reduction in iNOS accumulation, substantially dampening the pathological phenotype (**Figure 4b**). This protein-level effect was further corroborated by quantitative real-time PCR (qPCR) analysis of key activation markers. The classical pro-inflammatory co-stimulatory molecules, *CD80* and *CD86*, which were dramatically induced by LPS exposure, showed marked downregulation following DR treatment (**Figures 4c, d**). Notably, DR markedly suppressed these effector genes. In an additional qPCR experiment, MLA co-treatment did not abolish the DR-associated expression pattern of *Il1b, Il6*, and *Tnf* under the tested conditions (**Supplementary Figure S5**). Accordingly, this result was not interpreted as evidence of α7nAChR dependence. Together, these phenotypic data are consistent with attenuation of LPS-driven inflammatory effector programs by DR, and align with the RNA velocity patterns suggesting reduced vector orientation toward stress/inflammatory regions.

To delineate the treatment-associated transcriptional patterns accompanying these marker changes, we evaluated pseudotemporal expression patterns and RNA velocity. Along the inferred LPS-associated pseudotemporal ordering, cells showed increased expression of an interferon/stress response exemplified by Rsad2, Zfand5, and Vim. This ordering also included canonical inflammatory and integrated-stress modules, including Nos2 and Ddit3(**Figure 4e**), together with elevated elevated chemokine/stress-effector genes such as Cxcl2 and Saa3. Together, these observations are consistent with inferred ordering linking stress-response and inflammatory effector programs.

To move beyond static snapshots and characterize local transcriptional directionality, we performed RNA velocity analysis using vendor-provided spliced/unspliced matrices (loom) and scVelo’s dynamical model (**Figure 4f**). In control cells, velocity vectors formed a relatively continuous flow field, consistent with continuous organization across *Itgae^+^/Plk4^+^-*enriched regions of the UMAP space. In contrast, LPS substantially altered the velocity landscape, producing a strong bias of vectors toward stress/inflammatory regions—particularly regions enriched for Cxcl2+ and Saa3+ programs—indicating an LPS-associated inflammatory-related velocity pattern.

Intervention altered this velocity pattern, but in a drug-specific manner. DIO and DR were associated with an expression pattern characterized by the emergence of negative-feedback and recovery-associated programs, including *Il1rn* and *Dusp5*, alongside proteostasis and stress-recovery factors such as *Ppp1r15a*, *Bag3*, and *Hspa8*. In the velocity field, these treatments were associated with reduced orientation toward inflammatory regions and greater orientation toward *Itgae^+^/Plk4^+^*-enriched regions, with DR-specific patterns included in **Figure 4f**. This pattern is consistent with a feedback- and stress-adaptation-associated velocity pattern accompanied by reduced expression of inflammatory programs.

By contrast, GAL induced a distinct transcriptional pattern with prominent enrichment of protection/repair-associated genes such as *Neil3*, *Fancd2*, *Mcm6*, and *Prc1*, consistent with activation of DNA maintenance and repair programs. Thus, GAL and DR appear to represent two distinct intervention-associated transcriptional patterns: a genome-maintenance/repair-associated program under GAL, and a feedback- and stress-adaptation-associated program under DIO/DR.

### 3.4 Treatment-associated marker changes are accompanied by distinct TF regulon activity patterns

**Figure 5:**
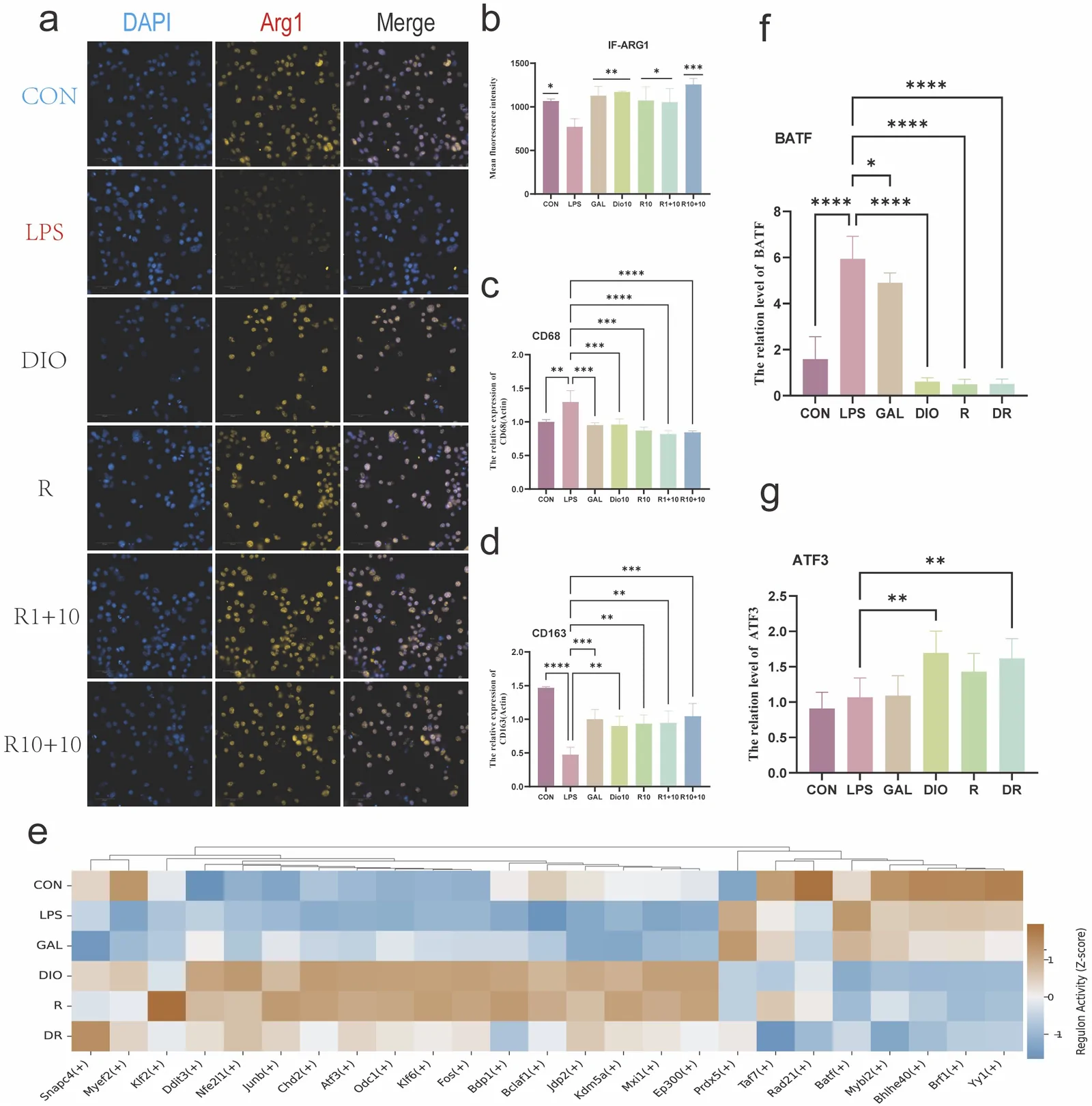
Distinct regulon-activity patterns accompany treatment-associated marker changes. treatment-associated changes in inflammatory and metabolic markers. **(a)** Representative immunofluorescence images of *Arg1* (arginine-metabolism-associated marker, red) in BV2 cells. Nuclei were stained with DAPI (blue). **(b)** Quantification of mean fluorescence intensity showing upregulation of *Arg1* in the combination treatment groups. **(c, d)** qPCR analysis of *CD68* **(c)** and *CD163* **(d)**, showing expression changes consistent with a treatment-associated changes in Cd68 and Cd163 expression. **(e)** SCENIC regulon activity heatmap. The DR group showed reduced regulon activity of the inflammation-associated program (Batf) and increased activities of Atf3- and Nfe2l1-associated cellular-maintenance/stress-buffering programs, together with a Ddit3-associated stress/ISR program, distinct from the patterns observed in the LPS and GAL groups. Color scale represents relative AUC scores; regulons shown passed the default NES threshold during network inference. **(f, g)** Quantitative real-time PCR measurement of Batf **(f)** and Atf3 **(g)** transcript levels. Replicate-level mean AUC values for selected regulons are shown in **Supplementary Figure S6**, with each point representing one biological replicate. Statistical comparisons in panels b–d and f–g were performed using ordinary one-way ANOVA followed by Tukey’s multiple-comparisons test. Each group contained three independent biological replicates (n = 3). Imaging fields were aggregated within each biological replicate, and qPCR technical duplicates were averaged before statistical analysis. Panel e is a descriptive SCENIC regulon-activity heatmap. Data are presented as mean ± SD. *P < 0.05, **P < 0.01, ***P < 0.001, ****P < 0.0001.

To interrogate the transcriptional logic underlying the treatment-associated marker changes described above, we performed SCENIC regulon inference and subsequently examined the transcript-level consistency of selected transcription factors by qPCR. Consistent with the feedback- and stress-adaptation-associated trajectory observed above, high-content imaging showed that Arg1 MFI was reduced by LPS and restored to varying degrees by treatment (**Figures 5a–d**). To interrogate candidate transcriptional associations accompanying these treatment-associated marker changes, we inferred transcription factor (TF) regulon activities using SCENIC. qPCR analyses of additional activation markers (e.g., *Cd80/Cd86*) and the scavenger-receptor-associated marker *Cd163* provided convergent support for treatment-driven phenotypic rebalancing, while Cd68 was interpreted as an activation/phagolysosomal program marker rather than a strict M1/M2 discriminator.

As illustrated in the heatmap (**Figure 5e**), LPS stimulation did not uniformly activate all AP-1 components; rather, it significantly suppressed a cluster of constitutively active immediate-early and homeostatic regulons, including *Junb, Fos, Atf3, Klf6*, and *Odc1*. Concurrently, LPS was associated with increased regulon activity of a distinct inflammatory program involving TFs such as Batf, Bhlhe40, and Mybl2. Notably, Batf has recently been implicated as a transcriptional driver of microglial hyperactivation and neurotoxicity (37). In inflammatory microglia, Batf has been reported to coordinate a pro-inflammatory gene program. Its elevated regulon activity under LPS in our dataset is consistent with a shift toward a more sustained inflammatory transcriptional state.

Treatment was associated with changes in the regulon-activity landscape. Importantly, the pharmacological comparator GAL and the experimental treatments (DIO, DR) showed different regulatory patterns. DIO and the combination treatment (DR) attenuated the LPS-associated inflammatory signature. They were associated with increased activity of Atf3- and Nfe2l1-associated cellular maintenance and stress buffering programs, together with a Ddit3-associated stress/ISR program, while simultaneously reducing the Batf-associated inflammatory module relative to LPS. Conceptually, this supports a feedback- and stress-adaptation-associated pattern in which the DR combination may attenuate the Batf-associated transcriptional program that contributes to the stressed inflammatory state, while showing increased activity of feedback- and stress-adaptation-associated programs (Atf3/Nfe2l1) that support remodeling of that state. In this framework, the differential engagement of Batf-versus Atf3-centered regulon programs represents a candidate transcriptional pattern associated with the distinction between persistent inflammatory states and recovery-associated remodeling. These regulatory inferences are consistent with the observed rebalancing of the *Nmur1*^+^/*Limk2*^+^–*Itgae*^+^ compositional axis and with the functional iNOS/Arg1 phenotypic recovery. Replicate-level mean AUC values showed consistent treatment-associated patterns for the Atf3(+), Batf(+), Ddit3(+), and Nfe2l1(+) regulons (**Supplementary Figure S6**). To examine transcript-level consistency with these computational predictions, we assessed the mRNA expression of these core transcription factors. Consistent with the regulon inferences, qPCR analysis showed that Batf mRNA expression was elevated by LPS and reduced under DR treatment (**Figure 5f**), while Atf3 expression was increased under DIO and DR treatments (**Figure 5g**). These transcript-level observations are consistent with the SCENIC-inferred Batf–Atf3-associated transcriptional pattern, though causal validation by TF perturbation experiments will be required to establish functional roles.

### 3.5 Neurotrophic-state enrichment links to BDNF/GDNF restoration

Finally, we examined whether state remodeling translated into recovery of neuroprotective function. ELISA showed that LPS significantly suppressed secretion of BDNF and GDNF, whereas DR effectively rescued both factors (**Figures 2a, b**). Single-cell module scoring indicated that this recovery was not a uniform population-wide increase in neurotrophic gene expression. Instead, neurotrophic scores exhibited a right-shifted, long-tailed distribution, consistent with an increased representation of cells with elevated neurotrophic scores (**Figure 2c**).

Given that the density distribution of neurotrophic scores exhibited a bimodal pattern with a right-skewed long tail (**Figure 2c**), we established an empirical threshold (score > 0.05) to exclude the dominant background (non-expressing) peak centered around zero, thereby specifically quantifying cells within the upper-score tail. Based on this threshold, we observed an increased fraction of neurotrophic-positive cells under DR treatment compared with LPS alone (**Figure 2d**). A similarly elevated fraction was observed in the R-only group. The upper neurotrophic-score distribution was additionally examined using sample-specific three-component GMMs as an alternative operational definition. The GMM-defined score-high fraction was 4.51% in the LPS group and 6.01% in the DR group, with similar values across the GAL, DIO, R, and DR groups (**Supplementary Figure S7** and **Supplementary Table 9**). All 18 models converged and showed clear component separation (Ashman’s D > 2). Because the fixed score >0.05 threshold and the GMM definition identified different fractions of cells, the GMM was interpreted descriptively rather than as validation of the fixed threshold. Furthermore, to rigorously dissect these phenotypes, we applied a stringent state classification framework. As illustrated by the pooled cellular landscape (**Figure 2e**), this approach successfully delineated the population into distinct functional states—including ‘neurotrophic only’ and ‘double positive’ subsets—highlighting the discrete presence of neurotrophic-prioritized programs amidst the broader inflammatory or double-negative background.

At the network-association level, co-expression network analysis linked *Bdnf*/*Gdnf* with *Igf1* and other repair-associated nodes, suggesting a cooperative neurotrophic module rather than isolated gene upregulation (**Figure 2f**). Regulatory-association analyses highlighted Atf3 and Jun as transcription factor-associated candidates and Igf1 as a signaling/network-associated node linked to neurotrophic-state enrichment, suggesting potential associations between stress-response circuitry and neurotrophic gene programs (**Figure 2g**). Together, these findings support a two-layer DR-associated response: (i) attenuation of inflammatory programs accompanied by rebalancing of marker-defined states, and (ii) an increased representation of cells with elevated neurotrophic scores accompanying restored BDNF and GDNF secretion (38).

## 4 Discussion

In this study, we compared the transcriptional and functional responses of LPS-challenged BV2 cells to (R)-nicotine, diosmetin, their combined administration (DR), and galantamine. Integration of scRNA-seq, RNA velocity, SCENIC inference, and functional measurements identified treatment-associated differences in marker-defined state distributions, inflammatory outputs, neurotrophic factors, and regulon activity. The DR condition was associated with attenuation of LPS-induced inflammatory responses without a significant reduction in CCK-8 signal. However, these findings do not establish restoration of physiological microglial homeostasis or a receptor-dependent mechanism.This distinction may have clinical relevance. Recent longitudinal studies emphasize that strategies indiscriminately depleting microglia disrupt essential CNS homeostasis, impair synaptic pruning, and ultimately exacerbate long-term neurological deficits (39, 40).

Cross-sectional compositional analysis identified the *Nmur1^+^/Limk2^+^* cluster as an intervention-sensitive marker-defined state, with DR treatment associated with a lower relative abundance of this cluster and a higher *Itgae^+^/Plk4^+^* cluster proportion than observed under LPS alone. This observation is consistent with the emerging single-cell transcriptomic consensus that microglial responses are distributed across context-dependent transcriptional states rather than a binary M1/M2 framework (28, 41, 42). Recent human single-cell and single-nucleus studies have similarly identified multiple pathology-, region-, and context-dependent microglial states in neurodegenerative disease rather than a single linear transition between homeostatic and activated identities (43–45). Accordingly, the *Itgae^+^/Plk4^+^* and *Nmur1^+^/Limk2^+^* clusters identified here should be interpreted as BV2-specific marker-defined states rather than direct equivalents of *in vivo* human microglial subtypes. RNA velocity provided inferred local transcriptional directionality, but the cross-sectional single-time-point design does not demonstrate direct movement of individual cells between transcriptional states.

Building upon this cellular landscape, we identified distinct intervention-associated transcriptional patterns under GAL and DR treatment: a genome-maintenance/repair-associated pattern under galantamine and a feedback- and stress-adaptation-associated pattern under DR. Both treatments were associated with reduced inflammatory readouts, but their dominant transcriptional features differed. Galantamine was associated with a genome-maintenance/repair-associated transcriptional pattern, characterized by robust activation of genome maintenance and DNA repair programs. In the present dataset, DR was associated with a feedback- and stress-adaptation-associated transcriptional program characterized by concurrent attenuation of pro-inflammatory programs and partial rebalancing of marker-defined states, whereas GAL was associated with a distinct genome-maintenance/repair-associated program. These patterns do not establish innate immune tolerance or active inflammatory resolution, both of which require temporal and functional evidence beyond the present single-time-point dataset (46, 47). Recent reviews of the plant-derived compounds apigenin and anatabine similarly highlight their potential multitarget anti-inflammatory, antioxidant, and neuromodulatory actions, while emphasizing the need for further mechanistic validation (48, 49). The feedback- and stress-adaptation-associated transcriptional features associated with DR may be relevant to chronic neuroinflammatory disorders (50, 51). However, whether these transcriptional patterns translate into neuroprotective effects *in vivo* remain to be determined.

SCENIC regulon analysis identified a candidate Batf–Atf3 transcriptional pattern that was differentially engaged across treatment conditions. Batf is an AP-1 family member reported to promote chromatin accessibility at pro-inflammatory loci in macrophages (52, 53). In the present study, its regulon activity—as inferred by SCENIC—and its transcript-level expression were markedly reduced under DR treatment; whether this reflects direct chromatin-level remodeling in BV2 cells requires validation by ATAC-seq or ChIP-seq. Concurrently, Atf3 regulon activity and mRNA levels were increased under DR treatment, consistent with an association with stress- response and inflammatory-feedback programs.

This apparent “dual-regulation” pattern may distinguish DR from the more selective Atf3-associated response observed under GAL treatment. Atf3 has emerged as a stress-responsive transcriptional regulator within the integrated stress response and macrophage regulatory networks. It has been implicated in NF-κB/cytokine regulation, oxidative-stress responses, and mitochondrial homeostasis (54–56). The reciprocal Batf and Atf3 changes observed here represent a candidate transcriptional pattern associated with the DR response and warrant further mechanistic investigation.

Beyond inflammatory attenuation, we propose a ‘functional long-tail’ model to describe the heterogeneous recovery of neurotrophic-related outputs. Our functional data showed that the increased secretion of BDNF and GDNF under DR relative to LPS was not accompanied by a uniform, population-wide transcriptional increase (51). Rather, it is accompanied by an increased representation of cells with elevated neurotrophic scores. Furthermore, co-expression analysis suggested that this neurotrophic program shows limited overlap with the global inflammatory transcriptional response, consistent with a heterogeneous cellular response rather than a uniform population-wide activation. This observation adds to the growing body of evidence for microglial functional heterogeneity, and is consistent with recent single-cell transcriptomic findings demonstrating that specific microglial subsets contribute to neuroprotective, synapse-preserving, and anti-excitotoxic functions (57, 58). Co-expression network analysis identified Atf3, Jun, and Igf1 as candidate network-associated nodes linked to neurotrophic-state enrichment. Within this inferred network, Igf1 showed relatively high connectivity between inflammatory and neurotrophic gene clusters, consistent with a potential bridging role. The functional significance and causal relationships among these nodes remain to be experimentally established. These findings support further characterization of cells with elevated neurotrophic programs, rather than relying solely on changes in the global population average (59).

All experiments were conducted in BV2 cells, a murine microglial cell line that diverges from primary microglia in key transcriptional features. Specifically, BV2 cells show reduced expression of homeostatic microglial markers such as P2ry12, Tmem119, and Cx3cr1 compared with primary microglia (60, 61), which may limit the direct translatability of subpopulation annotations—particularly the *Itgae^+^/Plk4^+^* cluster—to *in vivo* contexts. Future validation in primary microglial cultures or *in vivo* LPS/AD mouse models is warranted.

Standard quality control was applied to exclude low-quality cells; however, ambient RNA contamination was not explicitly modeled using decontamination tools (e.g., SoupX or DecontX), and doublet detection was performed using standard filtering criteria only. These factors may introduce noise into subpopulation assignments, particularly for rare clusters such as the neurotrophic-enriched subset. The Batf–Atf3-associated transcriptional pattern and the neurotrophic co-expression network identified here are derived from computational inference (SCENIC regulon scoring and co-expression analysis) and have not been experimentally validated by TF perturbation, ChIP-seq, or reporter assays. Causal relationships between these regulatory programs and the observed phenotypic outcomes remain to be established (32, 34). scRNA-seq data were collected at a single post-treatment time point, precluding assessment of the temporal stability of drug-induced state remodeling. The temporal stability of the DR-associated velocity pattern and cluster redistribution requires evaluation using longitudinal sampling. Previous work identified diosmetin as a candidate α7nAChR positive allosteric modulator based on computational screening and calcium-signaling analyses (24). However, receptor-subtype selectivity and relevance to BV2 cells were not established in that study (3, 50). In the present work, MLA co-treatment did not abolish the DR-associated *Il1b, Il6*, and *Tnf* expression pattern under the tested conditions. Therefore, the current data do not establish α7nAChR dependence, and alternative receptor-dependent or receptor-independent mechanisms remain possible. Receptor-expression analysis, validated antagonist controls, and genetic perturbation would be required to determine receptor involvement.

In conclusion, our findings indicate that the combination of (R)-nicotine and diosmetin is associated with remodeling of LPS-challenged BV2 microglial-like states, characterized by inflammatory-program attenuation and partial recovery of neurotrophic outputs, rather than traditional broad immunosuppression. By delineating treatment-associated state distributions and candidate regulatory patterns—most notably the Batf–Atf3-associated transcriptional pattern—at single-cell resolution, this study provides a framework for further evaluation of these responses in primary microglia and *in vivo* neuroinflammatory models.

## Data Availability

Underlying data and analysis code are available from the corresponding author upon reasonable request.
