# Supplementary material for: Single-cell transcriptomics reveals distinct microglial state remodeling associated with the (R)-nicotine/diosmetin combination and galantamine in LPS-challenged BV2 cells
Source: Front Immunol. 2026 Sep 1;17:1901868. doi: 10.3389/fimmu.2026.1901868 (PMC13574639; doi:10.3389/fimmu.2026.1901868)
Supplement: Supplementary file 1 [file DataSheet1.pdf]

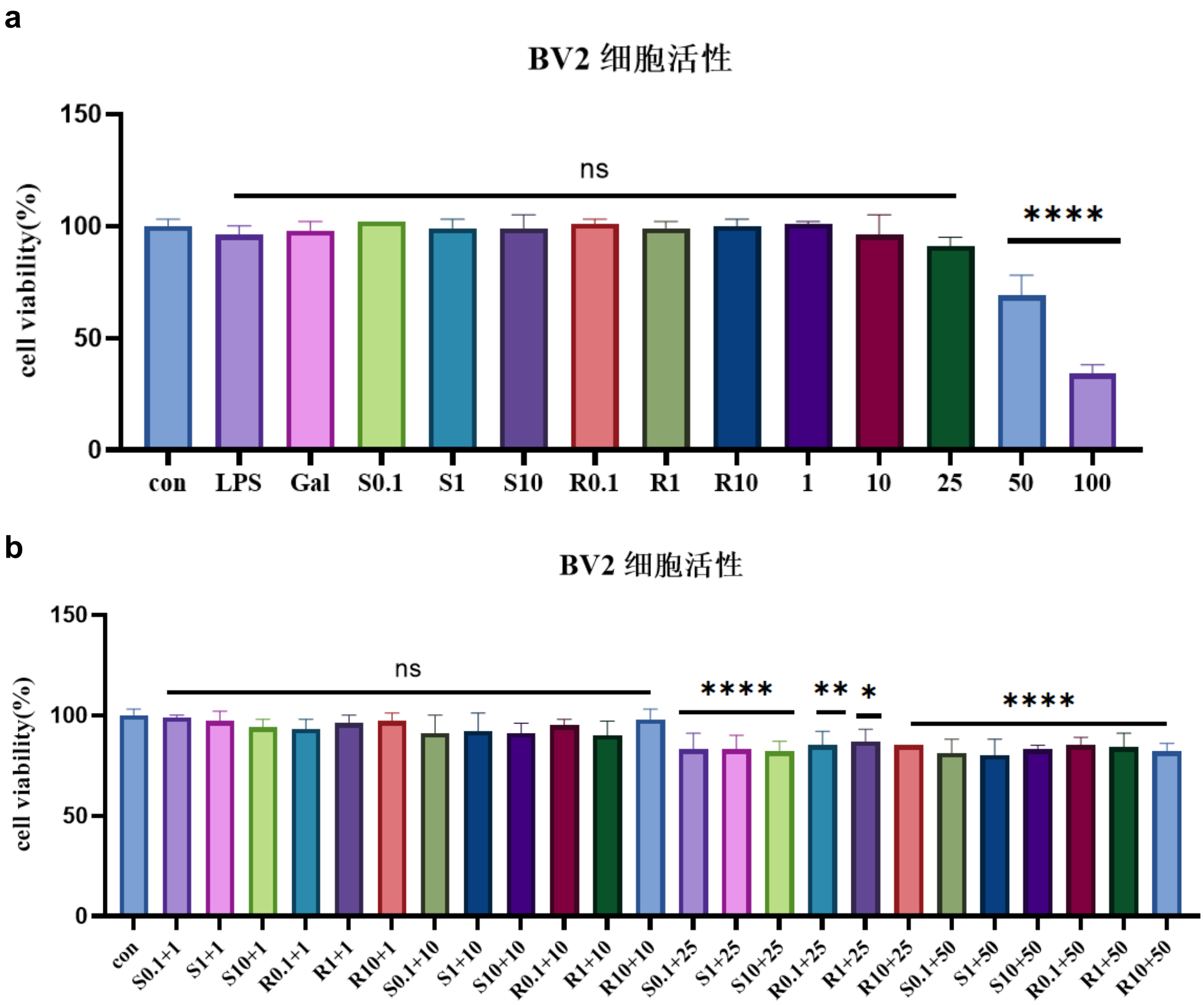


**Supplementary Figure 1. Cell viability assessment of BV2 cells under single-agent and combination treatments.(a)** Cell viability of BV2 cells following treatment with individual agents, including LPS, galantamine (GAL), diosmetin (S, at 0.1, 1, and 10 μM), and (R)-nicotine (R, at 0.1, 1, and 10 μM), as well as diosmetin alone at higher concentrations (1, 10, 25, 50, and 100 μM). All treatments except 50 μM and 100 μM diosmetin showed no significant cytotoxicity compared with the control (ns, not significant). Diosmetin at 50 μM and 100 μM significantly reduced cell viability (****, $p < 0.0001$), establishing the upper boundary for safe dosing.**(b)** Cell viability of BV2 cells following combination treatments of diosmetin and (R)-nicotine at various concentration ratios (S0.1+1, S1+1, S10+1, R0.1+1, R1+1, R10+1, S0.1+10, S1+10, S10+10, R0.1+10, R1+10, R10+10) and combinations with higher diosmetin concentrations (25 μM and 50 μM series). Combinations within the low-to-moderate concentration range showed no significant cytotoxicity (ns). However, combinations involving diosmetin at 25 μM (S0.1+25, S1+25, S10+25) and all combinations at the 50 μM diosmetin series showed significantly reduced cell viability (*, $p < 0.05$; **, $p < 0.01$; ****, $p < 0.0001$). These data informed the selection of non-cytotoxic concentration ranges (diosmetin ≤ 10 μM) for subsequent functional experiments.

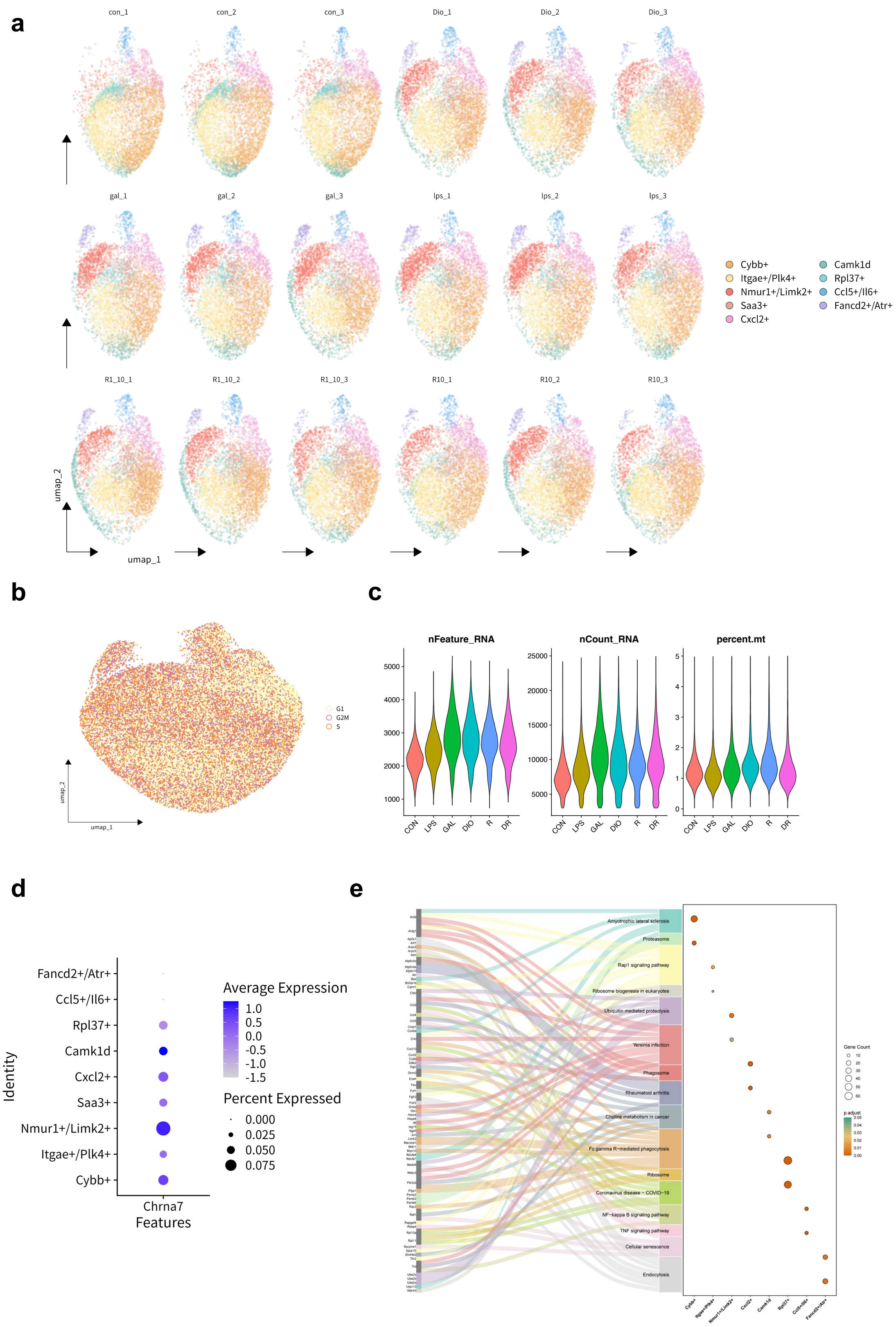


**Supplementary Figure 2. Quality control, replicate consistency, and functional enrichment of scRNA-seq data. (a)** UMAP embeddings split by individual biological replicates across all experimental conditions (n = 3 per group). The consistent cellular

distributions demonstrate high reproducibility and the absence of significant technical batch effects. **(b)** UMAP visualization of cell cycle phase scoring (G1, S, and G2M). Cell cycle-related variance was regressed out during data scaling to ensure that the defined microglial clusters reflect distinct functional states rather than mere proliferative status. **(c)** Post-filtering quality control (QC) metrics. Violin plots display the distributions of detected gene numbers (nFeature_RNA), total unique molecular identifiers (nCount_RNA), and the percentage of mitochondrial transcripts (percent.mt), indicating uniform, high-quality data across all experimental groups. **(d)** Dot plot showing the expression levels and expression frequency of the *Chrna7* gene (encoding the α7nAChR receptor) across the identified microglial subpopulations, validating the presence of the pharmacological target. **(e)** KEGG pathway enrichment analysis. The Sankey diagram maps the identified microglial clusters to their top differentially expressed marker genes and the correspondingly enriched biological pathways, supporting the functional annotation of each cellular state.
