# Supplementary material for: Single-cell transcriptomics reveals distinct microglial state remodeling associated with the (R)-nicotine/diosmetin combination and galantamine in LPS-challenged BV2 cells
Source: Front Immunol. 2026 Sep 1;17:1901868. doi: 10.3389/fimmu.2026.1901868 (PMC13574639; doi:10.3389/fimmu.2026.1901868)
Supplement: Supplementary file 2 [file DataSheet2.docx]

**Supplementary Figure S2. Replicate-level signature profiles across marker-defined BV2 clusters**. Box plots show sample × cluster mean scores for (A) the homeostatic microglial signature, (B) the inflammatory/NF-κB signature, (C) the stress/integrated stress response signature, and (D) the proliferation/cell-cycle signature. Signature scores were calculated at the single-cell level using AddModuleScore and subsequently averaged within each sample–cluster combination. Each point represents one biological sample–cluster combination. Overall differences among clusters were assessed using Friedman tests with sample identity as the blocking factor. The signatures were interpreted as relative and non-exclusive functional programs rather than definitive cluster identities.

**Supplementary Figure S3. Replicate-aware expression profiles of genes included in the four curated signatures.**Dot plots show expression of genes included in the homeostatic microglial, inflammatory/NF-κB, stress/ISR, and proliferation/cell-cycle signatures across the nine marker-defined clusters. Dot size represents the percentage of cells expressing each gene, and color represents scaled mean expression. Mean expression and detection frequency were first calculated within each sample–cluster combination and subsequently summarized across the 18 biological samples with equal sample weighting.

**Supplementary Figure S4. Replicate-level RNA velocity maps across the 18 biological samples.** RNA velocity streamlines were generated independently for the three biological replicates of each experimental condition. The maps are presented for CON, LPS, GAL, DIO, R, and DR. The three DR replicate-specific velocity objects contained 1,828, 1,907, and 1,873 cells, respectively. Replicate-level maps were used to assess the consistency of local vector organization and were not interpreted as direct lineage transitions.

**Supplementary Figure S5 Effect of MLA co-treatment on DR-associated gene expression.** Relative mRNA expression of *Il1b*, *Il6*, and *Tnf* was determined by qPCR in the CON, LPS, DR, and DR+MLA groups. MLA was used at 10 μM. Statistical comparisons were performed using ordinary one-way ANOVA followed by Tukey’s multiple-comparisons test. Data represent three independent biological replicates per group (n = 3), with technical duplicate measurements averaged within each biological replicate. P values were adjusted using the Benjamini–Hochberg method. Adjusted P values are indicated as *P < 0.05, **P < 0.01, ***P < 0.001, and ****P < 0.0001.

**Supplementary Figure S6. Replicate-level activities of selected treatment-associated regulons.** Regulon activity was calculated at the single-cell level using AUCell and subsequently averaged within each biological sample. Box plots show sample-level mean AUC values for the Atf3(+), Batf(+), Ddit3(+), and Nfe2l1(+) regulons across treatment groups. Each point represents one independent biological replicate (n = 3 per group).

**Supplementary Figure S7. Group-level summary of the sample-specific Gaussian-mixture sensitivity analysis.** Three-component Gaussian mixture models were fitted independently to each biological sample. Cells with a posterior probability >0.5 of belonging to the highest-mean component were classified as GMM-defined score-high cells. Bars show the group mean ± SD of the sample-specific score-high fractions, with each point representing one independent biological replicate (n = 3 per group). Model-specific cutoffs, highest-mean component parameters, and diagnostic results are provided in Supplementary Table S9.
